# Survival after recurrence in patients with gastric cancer who receive S-1 adjuvant chemotherapy: exploratory analysis of the ACTS-GC trial

**DOI:** 10.1186/s12885-018-4341-6

**Published:** 2018-04-20

**Authors:** Seiji Ito, Yasuo Ohashi, Mitsuru Sasako

**Affiliations:** 1 Department of Gastroenterological Surgery, Aichi Cancer Center, 1-1 Kanokoden, Chikusa-ku, Nagoya, Aichi 464-8681 Japan; 2 Department of Integrated Science and Engineering for Sustainable Society, Chuo University, 1-13-27 Kasuga, Bunkyo-ku, Tokyo, 112-8551 Japan; 3 Department of Surgery, Hyogo College of Medicine, 1-1 Mukogawa-cho, Nishinomiya City, Hyogo 663-8501 Japan

**Keywords:** ACTS-GC, Adjuvant chemotherapy, S-1, Recurrent gastric cancer, Stomach neoplasms

## Abstract

**Background:**

Some patients develop recurrence after curative resection and adjuvant chemotherapy. S-1, an oral fluoropyrimidine, is one of the standard regimens in adjuvant chemotherapy, and is also used in first-line treatment for advanced/metastatic gastric cancer. It is controversial as to whether the same treatment strategy can be applied for patients who develop recurrence after adjuvant chemotherapy and those who did not receive adjuvant chemotherapy. To investigate this issue, we compared the outcomes of patients who developed recurrences after treatment with or without adjuvant chemotherapy using the results of the Adjuvant Chemotherapy Trial of S-1 for Gastric Cancer (ACTS-GC).

**Methods:**

Patients who had confirmed recurrence in the ACTS-GC trial were analyzed. We defined 2 independent cohorts. Cohort 1 patients were divided by whether they received adjuvant chemotherapy (adjuvant S-1 group and surgery-only group). Cohort 2 patients were divided by whether they received a regimen including S-1 (IS) or not including S-1 (NIS) after recurrence.

**Results:**

A total of 375 patients experienced recurrence (160 in the adjuvant S-1 group and 215 in the surgery-only group). In cohort 1, the median time from recurrence to death (TFRD) was 11.4 months (95% confidence interval [CI], 8.4–13.9) in the adjuvant S-1 group and 11.3 months (95% CI, 9.7–13.1) in the surgery-only group (hazard ratio [HR], 1.05; 95% CI, 0.84–1.31). In cohort 2, 292 patients received chemotherapy after recurrence and were divided into the IS (*n* = 189) or the NIS group (*n* = 103). The median TFRD was 13.9 months (95% CI, 12.7–15.6) in the IS group and 8.1 months (95% CI, 6.6–9.7) in the NIS group (HR, 0.59; 95% CI, 0.45–0.76), and there was no significant interaction between the adjuvant S-1 group and surgery-only group.

**Conclusion:**

Adjuvant chemotherapy with S-1 prolonged overall survival without influencing the TFRD. The same treatment strategy may be applied for patients who develop recurrence after adjuvant chemotherapy and those who did not receive adjuvant chemotherapy.

**Trial registration:**

NCT00152217. First Posted on September 9, 2005.

**Electronic supplementary material:**

The online version of this article (10.1186/s12885-018-4341-6) contains supplementary material, which is available to authorized users.

## Background

Gastric cancer is the third leading cause of cancer-related death [1] and there were 723,100 deaths from gastric cancer worldwide in 2012. The mainstay of treatment for gastric cancer is surgery. In addition to surgery, clinical trials have shown that preoperative and/or postoperative adjuvant treatment is beneficial for potentially curable gastric cancer, although different approaches are used in the USA, European Union, and Asia [2–9].

The results of the Adjuvant Chemotherapy Trial of S-1 for Gastric cancer (ACTS-GC), a Japanese trial evaluating the effectiveness of 1 year of postoperative treatment with S-1, showed a significant survival benefit over surgery alone for patients with stage II or III gastric cancer according to the Japanese Classification of Gastric Carcinoma (Second English Edition) [10].

S-1 (Taiho Pharmaceutical, Tokyo, Japan) is an oral fluoropyrimidine that combines tegafur, a prodrug of 5-fluorouracil with gimeracil and oteracil potassium in a molar ratio of 1:0.4:1 [11]. The 5-year overall survival (OS) rate, the primary endpoint of the ACTS-GC at 5 years was 71.7% in the adjuvant S-1 group and 61.1% in the surgery-only group, demonstrating the usefulness of postoperative adjuvant chemotherapy with S-1 (hazard ratio [HR], 0.67; 95% confidence interval [CI], 0.54–0.83) [12, 13]. The relapse-free survival rate at 5 years was 65.4% in the adjuvant S-1 group and 53.1% in the surgery-only group (HR, 0.65; 95% CI, 0.54–0.79). On the basis of these results, S-1 is currently used as standard postoperative adjuvant chemotherapy for gastric cancer in Japan and Asian countries. However, approximately 35% of patients still develop recurrences after curative resection and adjuvant chemotherapy.

In colorectal, ovarian, and breast cancer, the strategy of reuse of the same drug as that used for postoperative adjuvant chemotherapy has been studied in patients who had postoperative recurrences (colorectal cancer: ACCENT [14], MOSAIC [15, 16], and NSABP C-07 [17, 18]; ovarian cancer: Markman et al. [18], ICON4/AGO-OVAR-2.2 [19], Harries et al. [20] and Fung et al. [21]; and breast cancer: RHEA [22]). In these cancers, similar to gastric cancer, the same drugs are used for adjuvant chemotherapy and for advanced/metastatic cancer treatment. In Asian and some European countries, S-1 is used as adjuvant chemotherapy and also as first-line treatment for advanced/metastatic gastric cancer. It is unclear whether regimens including S-1 show similar efficacy in those who develop recurrence after S-1 adjuvant chemotherapy as those who are naïve to S-1. Thus, we performed this exploratory analysis using the results of the ACTS-GC trial to investigate the duration of survival after recurrence comparing those who previously received a regimen including S-1 with those had not received S-1.

## Methods

### Patients, treatments, and follow-up

Patients were randomly assigned to either the adjuvant S-1 group or the surgery-only group in the ACTS-GC. The institutions participated in the ACTS-GC were shown in Additional file 1: Text S1. The main inclusion criteria were as follows: age 20–80 years, histologically confirmed stage II, IIIA, or IIIB after curative surgery, D2 or more extensive lymph node dissection with R0 surgery, adequate oral intake, and preserved major organ function. Tumor stage classification and D classification were in accordance with the Japanese Classification of Gastric Carcinoma (Second English Edition) [10]. The detailed inclusion and exclusion criteria were described previously [12].

Patients assigned to the adjuvant S-1 group received S-1 as adjuvant chemotherapy. S-1 was orally administered at a dose corresponding to the patient’s body surface area (BSA) (40 mg with BSA <  1.25 m^2^; 50 mg with BSA 1.25–1.5 m^2^; 60 mg with BSA > 1.5 m^2^) twice daily after meals for 28 consecutive days, followed by a 14-day rest period. Treatment was continued for 1 year after surgery.

Patients assigned to the surgery-only group received no anticancer treatment postoperatively until recurrence was confirmed either clinically or with imaging studies, which included ultrasonography, computed tomography, gastrointestinal radiography, and endoscopy. Every patient was followed up for 5 years after the date of surgery or death. Those who were alive at 5 years were censored at this point. Treatment after recurrence was not specified in the original protocol. Further details have been reported previously [12, 13]. Patients who had confirmed diagnosis of recurrent gastric cancer were included in this analysis.

### Cohort definition

We defined 2 different cohorts. In cohort 1, patients were divided by whether they received adjuvant chemotherapy. This cohort was used to investigate whether there were any differences in survival after recurrence between patients in the S-1 and surgery alone arm.

Cohort 2 patients were divided by whether they received a regimen including S-1 (IS) or not including S-1 (NIS) after recurrence. This cohort was used to investigate whether the efficacy of reuse of S-1 after recurrence was the same in those who received S-1 as adjuvant chemotherapy and those who were completely chemotherapy naïve. The IS group consisted of patients who received S-1-based chemotherapy for treatment of recurrent gastric cancer.

Patients assigned to the adjuvant S-1 group were divided into 2 groups according to whether they had developed recurrent disease within 6 months after the completion of adjuvant chemotherapy with S-1 (including recurrence during postoperative adjuvant chemotherapy with S-1), or at 6 or more months after the completion of adjuvant chemotherapy. This classification was based on the fact that recurrent tumors within 6 months after termination of adjuvant chemotherapy showed a different response to chemotherapy regimens in some other cancers, such as ovarian cancer.

### Statistical analysis

Time from recurrence to death (TFRD) was defined as the interval from the date of recurrence to the date of death from any cause. Survival rates were estimated by the Kaplan-Meier method. A Cox proportional hazards model was used to calculate HRs. A subgroup analysis was performed for the factors that may influence the outcome. Multivariate survival analysis was performed using a Cox proportional hazards model after checking for validity of the proportional hazards assumption by plotting log-minus-log survival curves. The distributions of patient characteristics between the two groups were compared using chi-square test. Results were defined as statistically significant at *P* <  0.05. All statistical analyses were performed with SAS software version 9.1 (SAS Institute Inc., Cary, NC, USA).

## Results

### Patients

**Fig. 1:**
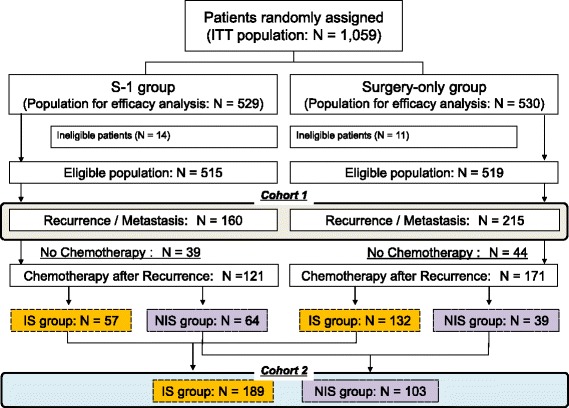
CONSORT diagram. Among eligible patients enrolled in the ACTS-GC, patients confirmed to have recurrence were included in this analysis. D1, D1 lymphadenectomy; ITT, intention to treat

Within 5 years after surgery, 375 out of 1034 eligible patients experienced recurrence (160 in the adjuvant S-1 group and 215 in the surgery-only group). Among these patients, 121 (75.6%) in the adjuvant S-1 group and 171 (79.5%) in the surgery-only group received chemotherapy after recurrence (Fig. 1). Eighty-three out of 375 received non-chemotherapeutic treatment (e.g., surgery, radiotherapy, or best supportive care: 39 in the adjuvant S-1 group and 44 in the surgery-only group). To treat the recurrence, S-1-based regimens were administered to 57 of 121 (35.6%) in the adjuvant S-1 group and 132 of 171 (61.4%) in the surgery-only group (*P* <  0.001) (Table 1).

**Table 1:** Patient characteristics in cohort 1

Cohort 1		S-1 adjuvant (*n* = 160)	Surgery only (*n* = 215)	Χ square test *p*-value
Sex	Male	111 (69.4)	148 (68.8)	0.911
	Female	49 (30.6)	67 (31.2)	
Age (years)^a^	< 60	45 (28.1)	67 (31.2)	0.577
	60–69	57 (35.6)	81 (37.7)	
	70–81	58 (36.3)	67 (31.2)	
Histologic type	Differentiated	62 (38.8)	74 (34.4)	0.345
	Undifferentiated	98 (61.3)	139 (64.7)	
	Others	0 (0.0)	2 (0.9)	
Cancer stage^b^ (Japanese Classification)	II	39 (24.4)	67 (31.2)	0.143
	IIIA	68 (42.5)	95 (44.2)	
	IIIB	53 (33.1)	53 (24.7)	
Site of first relapse^c^	Local	11 (6.9)	17 (7.9)	0.707
	Lymph nodes	30 (18.8)	53 (24.7)	0.173
	Peritoneum	76 (47.5)	97 (45.1)	0.647
	Hematogenous	59 (36.9)	69 (32.1)	0.334
Treatment after relapse				
Chemotherapy	Yes	121 (75.6)	171 (79.5)	0.367
	No	39 (24.4)	44 (20.5)	
Chemotherapy including S-1	Yes	57 (35.6)	132 (61.4)	< 0.001
	No	103 (64.4)	83 (38.6)	
Surgery	Yes	13 (8.1)	23 (10.7)	0.403
	No	147 (91.9)	192 (89.3)	
Radiotherapy	Yes	5 (3.1)	4 (1.9)	0.429
	No	155 (96.9)	211 (98.1)	

^a^Age at time of recurrence

^b^Cancer stage: Japanese Classification of Gastric Carcinoma (Second English Edition)

^c^Some patients had an initial recurrence at more than one site

### TFRD (adjuvant S-1 group and surgery-only group)

**Fig. 2:**
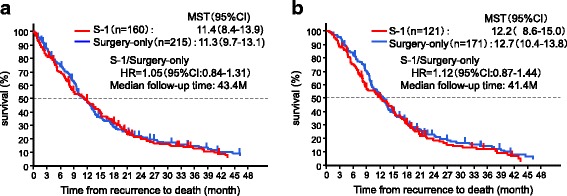
TFRD in cohort 1. TFRD among (**a**) all patients who had recurrent disease and (**b**) patients who received chemotherapy after the development of recurrent disease

The TFRD was analyzed according to the treatment group assigned at the time of enrollment in the ACTS-GC (adjuvant S-1 or surgery-only group) (Fig. 2a). The median duration of follow-up from recurrence was 43.4 months. The median TFRD was 11.4 (95% CI, 8.4–13.9) and 11.3 months in the adjuvant S-1 group (*n* = 160) and the surgery-only group (*n* = 215), respectively. The HR for death in the adjuvant S-1 group, as compared with the surgery-only group, was 1.05 (95% CI, 0.84–1.31). Among patients who received chemotherapy after the recurrence (adjuvant S-1 group, *n* = 121; surgery-only group, *n* = 171), the median duration of follow-up after recurrence was 41.4 months. The median TFRD was 12.2 months (95% CI, 8.6–15.0) in the adjuvant S-1 group and 12.7 months (95% CI, 10.4–13.8) in the surgery-only group. The HR for death in the adjuvant S-1 group, as compared with the surgery-only group, was 1.12 (95% CI, 0.87–1.44) (Fig. 2b). The regimens used for the patients who received chemotherapy after recurrences are shown in Additional file 2: Figure S1a.

### TFRD according to the chemotherapy regimen used after recurrence: IS vs. NIS

**Fig. 3:**
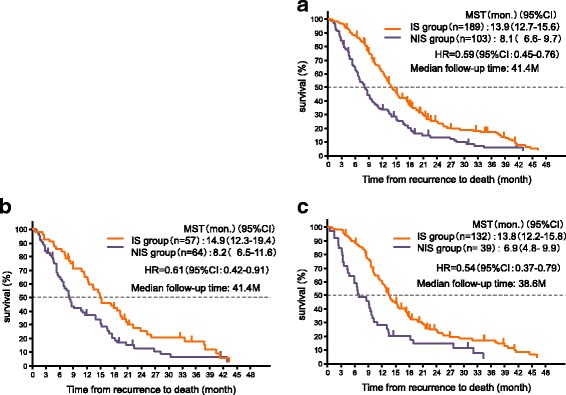
TFRD in cohort 2. TFRD for patients who received regimens (**a**) including S-1 and those who received regimens not including S-1 after recurrence (both treatment groups combined), (**b**) including S-1 and those who received regimens not including S-1 after recurrence (adjuvant S-1 group) and (**c**) including S-1 and those who received regimens not including S-1 after recurrence (surgery-only group)

**Fig. 4:**
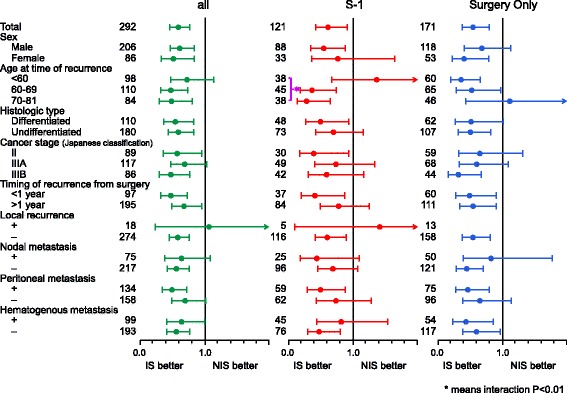
Subgroup analysis of the TFRD in each group. All groups, adjuvant S-1 group, and surgery-only group

The details of the IS or NIS groups are shown in Table 2. In both treatment groups combined, the median duration of follow-up after recurrence was 41.4 months. The median TFRD was 13.9 months (95% CI, 12.7–15.6) in the IS group (*n* = 189) and 8.1 months (95% CI, 6.6–9.7) in the NIS group (*n* = 103). The HR for death in the IS group, as compared with the NIS group, was 0.59 (95% CI, 0.45–0.76) (Fig. 3a). In the adjuvant S-1 group, the median duration of follow-up after recurrence was 41.4 months. The median TFRD was 14.9 months (95% CI, 12.3–19.4) in the IS group (*n* = 57) and 8.2 months (95% CI, 6.5–11.6) in the NIS group (*n* = 64). The HR for death in the IS group, as compared with the NIS group, was 0.61 (95% CI, 0.42–0.91) (Fig. 3b). In the surgery-only group, the median duration of follow-up from recurrence was 38.6 months. The median TFRD was 13.8 months (95% CI, 12.2–15.8) in the IS group (*n* = 132) and 6.9 months (95% CI, 4.8–9.9) in the NIS group (*n* = 39). The HR for death in the IS group, as compared with the NIS group, was 0.54 (95% CI, 0.37–0.79) (Fig. 3c). The TFRD was analyzed according to sex, age, histologic type, stage, timing of recurrence after surgery, and type of recurrence (Fig. 4). In subgroup analysis, there was no significant interaction between the adjuvant S-1 group and surgery-only group (*P* = 0.63, Additional file 3: Table S1). Moreover, the interactions between the IS and NIS groups were analyzed in both treatment groups combined, the adjuvant S-1 group and the surgery-only group. There was no significant interaction except for the age subgroup in the adjuvant S-1 group (*P* = 0.0059, Fig. 4). The outcomes were better in the IS group except for the age subgroups, irrespective of whether patients received S-1 as postoperative adjuvant chemotherapy.

**Table 2:** Patient characteristics in cohort 2

Cohort 2		IS group (*n* = 189)	NIS group (*n* = 103)	Χ square test *p*-value
Sex	Male	134 (70.9)	72 (69.9)	0.858
	Female	55 (29.1)	31 (30.1)	
Age (years)^a^	< 60	60 (31.8)	38 (36.8)	0.199
	60–69	68 (36.0)	42 (40.8)	
	70–81	61 (32.3)	23 (22.3)	
Histologic type	Differentiated	71 (37.6)	39 (37.9)	0.906
	Undifferentiated	117 (61.9)	63 (61.2)	
	Others	1 (0.5)	1 (1.0)	
Cancer stage^b^ (Japanese Classification)	II	64 (33.9)	25 (24.3)	0.226
	IIIA	73 (38.6)	44 (42.7)	
	IIIB	52 (27.5)	34 (33.0)	
Time from surgery to relapse (years)	< 1 y	56 (29.6)	41 (39.8)	0.078
	≥ 1 y	133 (70.4)	62 (60.2)	
Site of first relapse^c^	Local	16 (8.5)	2 (1.9)	0.027
	Lymph nodes	51 (27.0)	24 (23.3)	0.491
	Peritoneum	79 (41.8)	55 (53.4)	0.057
	Hematogenous	63 (33.3)	36 (35.0)	0.780

^a^Age at time of recurrence

^b^Cancer stage: Japanese Classification of Gastric Carcinoma (Second English Edition)

^c^Some patients had initial recurrence at more than one site

### TFRD according to the interval between completion of adjuvant chemotherapy and recurrence in the adjuvant S-1 group

The median TFRD was 13.4 months in the IS group and 6.8 months in the NIS group (HR, 0.57; 95% CI 0.30–1.08, *P* = 0.078) among patients with a recurrence-free interval (RFI) of less than 6 months. The median TFRD was 15.2 months in the IS group and 11.0 months in the NIS group (HR, 0.67; 95% CI 0.40–1.13, *P* = 0.13) among patients with an RFI of 6 months or longer. The 2 groups were not sufficiently large to allow definitive statistical conclusions, but the IS group seemed to have better OS after recurrence, irrespective of the interval between completion of adjuvant treatment and recurrence.

### Influence on survival after recurrence (multivariate analysis)

Among the eligible subjects, 375 patients who had recurrent gastric cancer (adjuvant S-1 group, *n* = 160; surgery-only group, *n* = 215) were included in multivariate Cox regression analysis to examine factors related to survival after recurrence (Additional file 4: Table S2). The HR for death was 0.64 (95% CI, 0.50–0.88, *P* < 0.001) among 225 patients who had recurrence more than 1 year after surgery (after completion of adjuvant treatment), as compared with 120 patients who had recurrence within 1 year after surgery (within adjuvant treatment periods), suggesting that the interval from surgery to recurrence is a determinant of the duration of survival after recurrence. The regimens used for patients who received chemotherapy after recurrence are shown in Additional file 2: Figure S1b, S1c.

## Discussion

Our study results suggest that the same treatment strategy can be applied to patients who develop recurrence after adjuvant chemotherapy and to those who have recurrence without adjuvant chemotherapy. To the best of our knowledge, similar studies, using results of phase III or meta-analysis, have not been performed in the field of gastric cancer. This analysis is the first investigation on this scale for gastric cancer. Two smaller retrospective reports [23, 24] have been previously published. Shitara et al. published a single-center and a multicenter retrospective study. In the single-center study, patients who developed recurrences after adjuvant chemotherapy with S-1 were retrospectively divided by whether they received S-1-containing regimen or non-S-1-containing regimen after recurrence. The non-S-1-containing regimen group had a high response rate and better progression-free survival than the S-1-containing regimen group [23]. In contrast, the multicenter report showed that S-1 plus cisplatin (SP) therapy, as first-line chemotherapy after recurrence, yielded excellent results in patients with an RFI of at least 6 months [24].

In our exploratory analysis of data from the ACTS-GC, patients who received adjuvant chemotherapy with S-1 tended to have a similar prognosis after recurrence as patients who did not receive adjuvant chemotherapy. In other words, prolonged OS in ACTS-GC study may directly reflect the relapse free survival (RFS) benefit of the adjuvant chemotherapy. This finding was consistent even when limiting the analysis to patients who received chemotherapy after recurrence.

Another clinical question is the selection of a regimen for patients with recurrence after adjuvant chemotherapy. Generally, as a principle of chemotherapy, reuse of the same drug is rarely considered for refractory patients. However, it is not clear whether the same strategy should be applied to patients with recurrence after adjuvant chemotherapy. Our analysis revealed that reuse of an S-1 including regimen after recurrence showed better results than a regimen without S-1 for all populations (both treatment groups combined, adjuvant S-1 group, and surgery-only group).

There were some limitations to our analysis; for example, some of the detailed information on patient characteristics such as performance status at recurrence and availability of oral administration was lacking, and thus, the effects of such factors could not be excluded. To minimize any bias, subgroup analysis was performed according to factors that may influence prognosis, including age at recurrence, timing of recurrence, and site of recurrence. We found the IS regimen had a better outcome than NIS for almost all subgroups. An interaction was found only in the subgroup of age at recurrence in the adjuvant S-1 group, although the difference was not significant (HR = 1.37, 95% CI 0.67–2.78). Thus, for patients younger than 60 years at the time of recurrence, a regimen not including S-1 should be considered.

The XParTS trial, a phase II trial with a small sample size, investigated the efficacy of reuse of fluoropyrimidine-based anticancer agents in patients receiving adjuvant chemotherapy with S-1, and its results suggested this regimen’s efficacy in patients with recurrence after adjuvant chemotherapy [25]. This finding supports our analysis results. Currently, a phase II trial (KSCC1001, UMIN ID: UMIN000004303) is investigating the efficacy of SP therapy in patients with gastric cancer who had recurrence at least 6 months after adjuvant chemotherapy with S-1, and a randomized phase II trial (OGSG1103, UMIN ID: UMIN000006105) is investigating the efficacy of SP and capecitabine plus cisplatin therapies in patients with gastric cancer who had recurrences after adjuvant chemotherapy with S-1. We may need to re-interpret our analysis results in the context of the results of these studies.

## Conclusions

Our analysis showed that postoperative adjuvant chemotherapy with S-1 for stage II or III gastric cancer prolonged recurrence-free survival without influencing the TFRD. Furthermore, our results suggest that S-1-based regimens may be effective for the management of patients with recurrent gastric cancer who received S-1 as adjuvant chemotherapy. Patients with recurrence after adjuvant chemotherapy with S-1 may have the same degree of sensitivity to chemotherapy for recurrent gastric cancer as those who develop recurrences without adjuvant chemotherapy. Thus, the same treatment strategy may be applied to patients who develop recurrences after adjuvant chemotherapy and those who did not receive adjuvant chemotherapy.

## Additional files

Additional file 1: Text S1. Institutions participating in the ACTS-GC. (DOCX 30 kb)

Additional file 2: **Figure S1.** Chemotherapeutic regimens used after recurrence. Chemotherapeutic regimens used after recurrence in (a) all recurrence patients, (b) patients who had recurrence within 1 year after surgery, and (c) patients who had recurrence more than 1 year after surgery. (DOCX 166 kb)

Additional file 3: **Table S1.** Subgroup analysis of the time from recurrence to death in each group (detail for Fig. 4). (DOCX 51 kb)

Additional file 4: **Table S2.** Multivariate Cox regression analysis of survival after recurrence. (DOCX 29 kb)

## Abbreviations

ACTS-GC: Adjuvant Chemotherapy Trial of S-1 for Gastric cancer
BSA: Body surface area
CI: Confidence interval
HR: Hazard ratio
IS: Including S-1
NIS: Not including S-1
OS: Overall survival
RFI: Recurrence-free interval
TFRD: Time from recurrence to death
