# Supplementary material for: Survival after recurrence in patients with gastric cancer who receive S-1 adjuvant chemotherapy: exploratory analysis of the ACTS-GC trial
Source: BMC Cancer. 2018 Apr 20;18:449. doi: 10.1186/s12885-018-4341-6 (PMC5910584; doi:10.1186/s12885-018-4341-6)
Supplement: Supplementary file 1 — Text S1. Institutions participating in the ACTS-GC. (DOCX 30 kb) [file 12885_2018_4341_MOESM1_ESM.docx]

**Text S1. Institutions participated in the ACTS-GC**

The following institutions participated in this study:

Sapporo Medical University Hospital (Hokkaido), Steel Memorial Muroran Hospital (Hokkaido), Hokkaido University Hospital (Hokkaido), Hirosaki University Hospital (Aomori), Iwate Medical University Hospital (Iwate), Iwate Prefectural Central Hospital (Iwate), National Hospital Organization Sendai Medical Center (Miyagi), Miyagi Cancer Center (Miyagi), Tohoku University Hospital (Miyagi), Yamagata Prefectural Central Hospital (Yamagata), Noshiro Kousei Medical Center (Akita), Fukushima Medical University Hospital (Surgery I; Fukushima), Fukushima Medical University Hospital (Surgery II; Fukushima), Tsuboi Cancer Center Hospital (Fukushima), Saiseikai Niigata Daini Hospital (Niigata), Niigata Cancer Center Hospital (Niigata), Gunma University Hospital (Gunma), Gunma Prefectural Cancer Center (Gunma), Tsuchiura Kyodo General Hospital (Ibaraki), Tsukuba University Hospital (Ibaraki), Dokkyo Medical University Hospital (Tochigi), Tochigi Cancer Center (Utsunomiya), Saitama Cancer Center (Saitama), Dokkyo Medical University Koshigaya Hospital (Saitama), Saitama Medical University Hospital (Saitama), Chiba University Hospital (Chiba), National Cancer Center Hospital East (Chiba), Chiba Rousai Hospital (Chiba), Chiba Cancer Center (Chiba), National Center for Global Health and Medicine (Tokyo), Showa University Koto Toyosu Hospital (Tokyo), National Cancer Center Hospital (Tokyo), Fraternity Memorial Hospital (Tokyo), Tokyo Metropolitan Bokutoh Hospital (Tokyo), Showa University Hospital (Tokyo), Tokyo Metropolitan Cancer and Infectious Diseases Center Komagome Hospital (Tokyo), Mitsui Memorial Hospital (Tokyo), Cancer Institute Hospital of JFCR (Tokyo), Nihon University Itabashi Hospital (Tokyo), Tokyo Metropolitan Tama Medical Center (Tokyo), Showa General Hospital (Tokyo), Keio University Hospital (Tokyo), National Hospital Organization Tokyo Medical Center (Tokyo), St. Luke's International Hospital (Tokyo), Medical Hospital, Tokyo Medical and Dental University (Tokyo), Kitasato University East Hospital (Kanagawa), Yokohama City University Medical Center (Kanagawa), Yokohama Municipal Citizen's Hospital (Kanagawa), Kanagawa Cancer Center (Kanagawa), Yokosuka Kyosai Hospital (Kanagawa), Yokohama Rosai Hospital (Kanagawa), Saiseikai Yokohamashi Nanbu Hospital (Kanagawa), Shizuoka General Hospital (Shizuoka), Fujieda Municipal General Hospital (Shizuoka), Showa Inan General Hospital (Nagano), Shinshu University Hospital (Nagano), Nagano Municipal Hospital (Nagano), Gifu Municipal Hospital (Gifu), Ogaki Municipal Hospital (Gifu), Chukyo Hospital (Aichi), Aichi Cancer Center Hospital (Aichi), National Hospital Organization Nagoya Medical Center (Aichi), Aichi Medical University Hospital (Aichi), Aichi Cancer Center Aichi Hospital (Aichi), Kainan Hospital (Aichi), Fukui-ken Saiseikai Hospital (Fukui), Japanese Red Cross Fukui Hospital (Fukui), Toyama Prefectural Central Hospital (Toyama), Kyoto Daini Red Cross Hospital (Kyoto), Japanese Red Cross Kyoto Daiichi Hospital (Kyoto), NTT West Osaka Hospital (Osaka), Osaka City General Hospital (Osaka), National Hospital Organization Osaka National Hospital (Osaka), Osaka Medical Center for Cancer and Cardiovascular Diseases (Osaka), Sakai City Medical Center (Osaka), Kinki University Hospital, Faculty of Medicine (Osaka), Osaka Red Cross Hospital (Osaka), Kansai Medical University Takii Hospital (Osaka), Minoh City Hospital (Osaka), Kobe University Hospital (Hyogo), Hyogo Cancer Center (Hyogo), Kansai Rosai Hospital (Hyogo), Hiroshima University Hospital (Hiroshima), National Hospital Organization Kure Medical Center and Chugoku Cancer Center (Hiroshima), Hiroshima City Asa Citizens Hospital (Hiroshima), Hiroshima Red Cross Hospital and Atomic-Bomb Survivors Hospital (Hiroshima), Shimane Prefectural Central Hospital (Shimane), Tottori University Hospital (Tottori), Okayama Saiseikai General Hospital (Okayama), Yamaguchi University Hospital (Yamaguchi), Tokushima University Hospital (Tokushima), National Hospital Organization Shikoku Cancer Center (Ehime), Matsuyama Red Cross Hospital (Ehime), National Kyushu Cancer Center (Fukuoka), National Hospital Organization Kyushu Medical Center (Fukuoka), Kitakyushu Municipal Medical Center (Fukuoka), Fukuoka University Chikushi Hospital (Fukuoka), Saiseikai Fukuoka General Hospital (Fukuoka), Kokura Memorial Hospital (Fukuoka), Tagawa Hospital (Fukuoka), Saga-ken Medical Center Koseikan (Saga), Sasebo City General Hospital (Nagasaki), National Hospital Organization Nagasaki Medical Center (Nagasaki), Miyazaki Prefectural Miyazaki Hospital (Miyazaki), Oita Prefectural Hospital (Oita), Saiseikai Kumamoto Hospital (Kumamoto), Kumamoto Regional Medical Center (Kumamoto), Japanese Red Cross Kumamoto Hospital (Kumamoto), National Hospital Organization Kumamoto Medical Center (Kumamoto).
