# Supplementary material for: Survival after recurrence in patients with gastric cancer who receive S-1 adjuvant chemotherapy: exploratory analysis of the ACTS-GC trial
Source: BMC Cancer. 2018 Apr 20;18:449. doi: 10.1186/s12885-018-4341-6 (PMC5910584; doi:10.1186/s12885-018-4341-6)
Supplement: Supplementary file 2 — Figure S1. Chemotherapeutic regimens used after recurrence. Chemotherapeutic regimens used after recurrence in (a) all recurrence patients, (b) patients who had recurrence within 1 year after surgery, and (c) patients who had recurrence more than 1 year after surgery. (DOCX 166 kb) [file 12885_2018_4341_MOESM2_ESM.docx]

Fig. S1. **Chemotherapeutic regimens used after recurrence**

(a) **All recurrence patients**


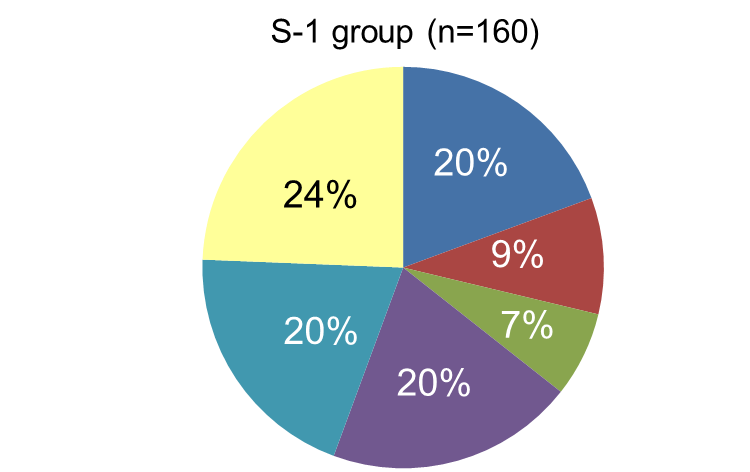

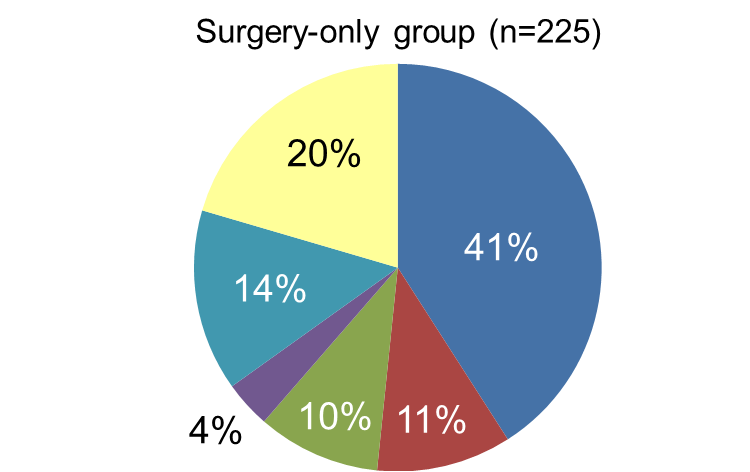


(b) **Patients who had recurrence within 1 year after surgery**


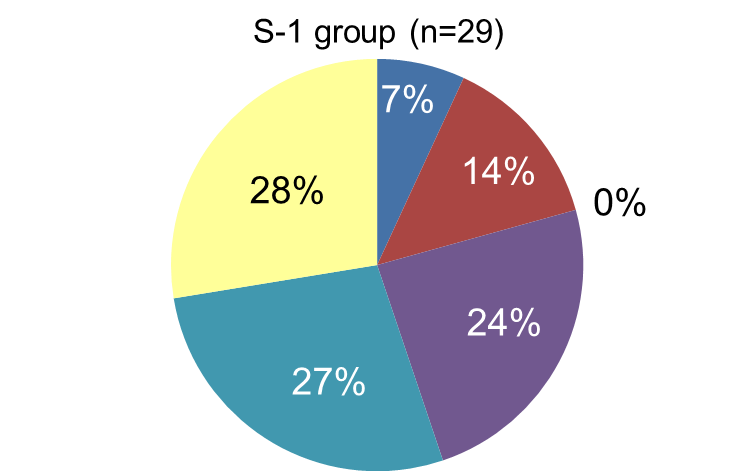

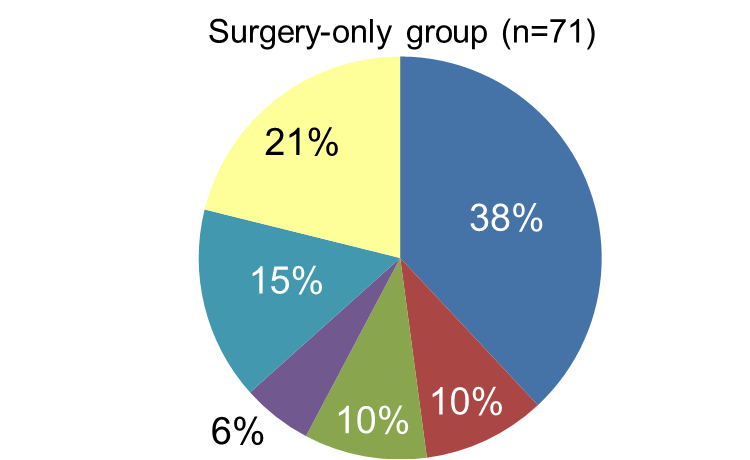


(c) **Patients who had recurrence more than 1 year after surgery**


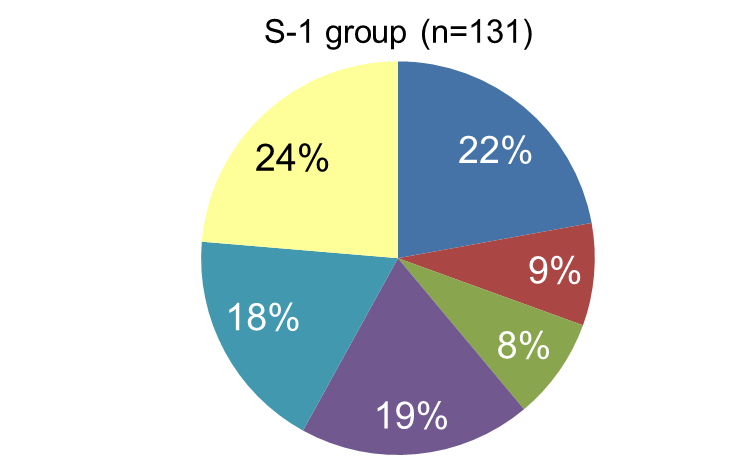

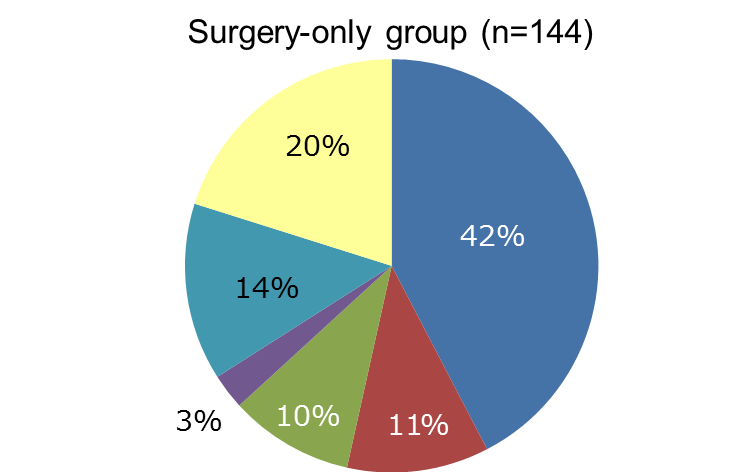


■S-1 alone　■S-1 plus cisplatin　■S-1+others　■Taxane alone　■Others (excluding S-1)　■None
