# Supplementary material for: Survival after recurrence in patients with gastric cancer who receive S-1 adjuvant chemotherapy: exploratory analysis of the ACTS-GC trial
Source: BMC Cancer. 2018 Apr 20;18:449. doi: 10.1186/s12885-018-4341-6 (PMC5910584; doi:10.1186/s12885-018-4341-6)
Supplement: Supplementary file 3 — Table S1. Subgroup analysis of the time from recurrence to death in each group (detail for Fig. 4). (DOCX 51 kb) [file 12885_2018_4341_MOESM3_ESM.docx]

Table S1. **Subgroup analysis of the time from recurrence to death in each group (detail for Figure. 4).**

|  |  | All group | | | |  |  |  |  |  | S-1 group | | | |  |  |  |  |  | Surgery-only group | | | | |  |  |  |
| --- | --- | --- | --- | --- | --- | --- | --- | --- | --- | --- | --- | --- | --- | --- | --- | --- | --- | --- | --- | --- | --- | --- | --- | --- | --- | --- | --- |
|  | | n | IS  group | NIS  group | HR | 95%CI | | | interaction  P |  | n | IS  group | NIS  group | HR | 95%CI | | | interaction  P |  | n | IS  group | NIS  group | HR | 95%CI | | | interaction  P |
| Total |  | 292 | 189 | 103 | 0.59 | 0.45 | - | 0.76 |  |  | 121 | 57 | 64 | 0.61 | 0.42 | - | 0.91 |  |  | 171 | 132 | 39 | 0.54 | 0.37 | - | 0.79 |  |
|  |  |  |  |  |  |  |  |  |  |  |  |  |  | Interaction^a^：P=0.634 | | | | | | | | | | | | |  |
|  |  |  |  |  |  |  |  |  |  |  |  |  |  |  |  |  |  |  |  |  |  |  |  |  |  |  |  |
| Sex | male | 206 | 134 | 72 | 0.613 | 0.450 | - | 0.834 | P=0.556 |  | 88 | 37 | 51 | 0.543 | 0.339 | - | 0.868 | P=0.459 |  | 118 | 97 | 21 | 0.676 | 0.408 | - | 1.117 | P=0.227 |
|  | female | 86 | 55 | 31 | 0.516 | 0.320 | - | 0.833 |  |  | 33 | 20 | 13 | 0.762 | 0.355 | - | 1.637 |  |  | 53 | 35 | 18 | 0.408 | 0.214 | - | 0.778 |  |
|  |  |  |  |  |  |  |  |  |  |  |  |  |  | Interaction^a^：P=0.560 | | | | | | | | | | | | |  |
|  |  |  |  |  |  |  |  |  |  |  |  |  |  |  |  |  |  |  |  |  |  |  |  |  |  |  |  |
| Age at time of  recurrence  (years) | ＜60 | 98 | 60 | 38 | 0.726 | 0.465 | - | 1.135 | P=0.336 |  | 38 | 19 | 19 | 1.365 | 0.670 | - | 2.783 | P=0.006 |  | 60 | 41 | 19 | 0.354 | 0.193 | - | 0.649 | P=0.141 |
|  | 60-69 | 110 | 68 | 42 | 0.477 | 0.311 | - | 0.730 |  |  | 45 | 18 | 27 | 0.355 | 0.173 | - | 0.730 |  |  | 65 | 50 | 15 | 0.523 | 0.283 | - | 0.967 |  |
|  | 70-81 | 84 | 61 | 23 | 0.482 | 0.290 | - | 0.800 |  |  | 38 | 20 | 18 | 0.284 | 0.127 | - | 0.635 |  |  | 46 | 41 | 5 | 1.108 | 0.426 | - | 2.885 |  |
|  |  |  |  |  |  |  |  |  |  |  |  |  |  | Interaction^a^：P=0.014 | | | | | | | | | | | | |  |
|  |  |  |  |  |  |  |  |  |  |  |  |  |  |  |  |  |  |  |  |  |  |  |  |  |  |  |  |
| Histologic type | Differentiated | 110 | 71 | 39 | 0.545 | 0.361 | - | 0.824 | P=0.778 |  | 48 | 21 | 27 | 0.494 | 0.263 | - | 0.930 | P=0.414 |  | 62 | 50 | 12 | 0.511 | 0.261 | - | 1.000 | P=0.979 |
|  | Undifferentiated | 180 | 117 | 63 | 0.589 | 0.421 | - | 0.823 |  |  | 73 | 36 | 37 | 0.693 | 0.418 | - | 1.148 |  |  | 107 | 81 | 26 | 0.505 | 0.314 | - | 0.814 |  |
|  |  |  |  |  |  |  |  |  |  |  |  |  |  | Interaction^a^：P=0.783 | | | | | | | | | | | | |  |
|  |  |  |  |  |  |  |  |  |  |  |  |  |  |  |  |  |  |  |  |  |  |  |  |  |  |  |  |
| Cancer stage  (Japanese　 classification) | II | 89 | 64 | 25 | 0.570 | 0.343 | - | 0.948 | P=0.488 |  | 30 | 17 | 13 | 0.385 | 0.160 | - | 0.926 | P=0.498 |  | 59 | 47 | 12 | 0.648 | 0.320 | - | 1.309 | P=0.324 |
|  | IIIA | 117 | 73 | 44 | 0.688 | 0.464 | - | 1.021 |  |  | 49 | 21 | 28 | 0.731 | 0.400 | - | 1.335 |  |  | 68 | 52 | 16 | 0.599 | 0.332 | - | 1.083 |  |
|  | IIIB | 86 | 52 | 34 | 0.472 | 0.293 | - | 0.761 |  |  | 42 | 19 | 23 | 0.592 | 0.303 | - | 1.157 |  |  | 44 | 33 | 11 | 0.320 | 0.152 | - | 0.670 |  |
|  |  |  |  |  |  |  |  |  |  |  |  |  |  | Interaction^a^：P=0.564 | | | | | | | | | | | | |  |
|  |  |  |  |  |  |  |  |  |  |  |  |  |  |  |  |  |  |  |  |  |  |  |  |  |  |  |  |

Table S1. **continued**

| Timing of recurrence from surgery | <1 year | 97 | 56 | 41 | 0.471 | 0.308 | - | 0.720 | P=0.186 |  | 37 | 12 | 25 | 0.408 | 0.195 | - | 0.856 | P=0.152 |  | 60 | 44 | 16 | 0.491 | 0.268 | - | 0.899 | P=0.799 |
| --- | --- | --- | --- | --- | --- | --- | --- | --- | --- | --- | --- | --- | --- | --- | --- | --- | --- | --- | --- | --- | --- | --- | --- | --- | --- | --- | --- |
|  | >1 year | 195 | 133 | 62 | 0.677 | 0.487 | - | 0.943 |  |  | 84 | 45 | 39 | 0.776 | 0.483 | - | 1.247 |  |  | 111 | 88 | 23 | 0.543 | 0.330 | - | 0.894 |  |
|  |  |  |  |  |  |  |  |  |  |  |  |  |  | Interaction^a^：P=0.444 | | | | | | | | | | | | |  |
|  |  |  |  |  |  |  |  |  |  |  |  |  |  |  |  |  |  |  |  |  |  |  |  |  |  |  |  |
| Local recurrence | + | 18 | 16 | 2 | 1.059 | 0.219 | - | 5.119 | P=0.462 |  | 5 | 3 | 2 | 1.414 | 0.085 | - | 23.573 | P=0.555 |  | 13 | 13 | 0 | - |  | - |  | - |
|  | – | 274 | 173 | 101 | 0.581 | 0.446 | - | 0.757 |  |  | 116 | 54 | 62 | 0.601 | 0.404 | - | 0.892 |  |  | 158 | 119 | 39 | 0.544 | 0.370 | - | 0.800 |  |
|  |  |  |  |  |  |  |  |  |  |  |  |  |  | Interaction^a^：P=0.772 | | | | | | | | | | | | |  |
|  |  |  |  |  |  |  |  |  |  |  |  |  |  |  |  |  |  |  |  |  |  |  |  |  |  |  |  |
| Nodal metastasis | + | 75 | 51 | 24 | 0.641 | 0.381 | - | 1.080 | P=0.640 |  | 25 | 10 | 15 | 0.432 | 0.172 | - | 1.088 | P=0.373 |  | 50 | 41 | 9 | 0.824 | 0.385 | - | 1.762 | P=0.169 |
|  | – | 217 | 138 | 79 | 0.556 | 0.411 | - | 0.750 |  |  | 96 | 47 | 49 | 0.688 | 0.444 | - | 1.067 |  |  | 121 | 91 | 30 | 0.443 | 0.283 | - | 0.694 |  |
|  |  |  |  |  |  |  |  |  |  |  |  |  |  | Interaction^a^：P=0.368 | | | | | | | | | | | | |  |
|  |  |  |  |  |  |  |  |  |  |  |  |  |  |  |  |  |  |  |  |  |  |  |  |  |  |  |  |
| Peritoneal metastasis | + | 134 | 79 | 55 | 0.493 | 0.339 | - | 0.716 | P=0.193 |  | 59 | 26 | 33 | 0.493 | 0.279 | - | 0.871 | P=0.320 |  | 75 | 53 | 22 | 0.459 | 0.267 | - | 0.791 | P=0.385 |
|  | – | 158 | 110 | 48 | 0.698 | 0.484 | - | 1.008 |  |  | 62 | 31 | 31 | 0.736 | 0.426 | - | 1.273 |  |  | 96 | 79 | 17 | 0.649 | 0.372 | - | 1.132 |  |
|  |  |  |  |  |  |  |  |  |  |  |  |  |  | Interaction^a^：P=0.594 | | | | | | | | | | | | |  |
|  |  |  |  |  |  |  |  |  |  |  |  |  |  |  |  |  |  |  |  |  |  |  |  |  |  |  |  |
| Hematogenous metastasis | + | 99 | 63 | 36 | 0.637 | 0.409 | - | 0.992 | P=0.625 |  | 45 | 23 | 22 | 0.811 | 0.431 | - | 1.525 | P=0.199 |  | 54 | 40 | 14 | 0.439 | 0.227 | - | 0.848 | P=0.457 |
|  | – | 193 | 126 | 67 | 0.556 | 0.404 | - | 0.764 |  |  | 76 | 34 | 42 | 0.477 | 0.288 | - | 0.791 |  |  | 117 | 92 | 25 | 0.597 | 0.373 | - | 0.954 |  |
|  |  |  |  |  |  |  |  |  |  |  |  |  |  | Interaction^a^：P=0.513 | | | | | | | | | | | | |  |
| ^a^ Interaction among three factors. The three factors were follows;  Patients characteristics  Type of adjuvant chemotherapy(S-1 or Surgery alone)  Chemotherapy after recurrence(IS or NIS) | | | | | | | | | | | | |  |  | | | | | | | | | | | | |  |
