# Supplementary material for: Survival after recurrence in patients with gastric cancer who receive S-1 adjuvant chemotherapy: exploratory analysis of the ACTS-GC trial
Source: BMC Cancer. 2018 Apr 20;18:449. doi: 10.1186/s12885-018-4341-6 (PMC5910584; doi:10.1186/s12885-018-4341-6)
Supplement: Supplementary file 4 — Table S2. Multivariate Cox regression analysis of survival after recurrence. (DOCX 29 kb) [file 12885_2018_4341_MOESM4_ESM.docx]

Table S2. **Multivariate Cox regression analysis of survival after recurrence**

| Characteristic | No of Pts | HR | 95% CI | | | P value |
| --- | --- | --- | --- | --- | --- | --- |
| Treatment group |  |  |  | | |  |
| Surgery only | 215 | 1  1.035 | 0.827 | - | 1.295 | 0.763 |
| S-1 | 160 |  |  |  |  |  |
| Sex |  |  | | | | |
| Male | 259 | 1  1.046 | 0.816 | - | 1.341 | 0.723 |
| Female | 116 |  |  |  |  |  |
| Age at recurrence (years) |  |  | | | | |
| <60 | 112 | 1  1.177 | 0.889 | - | 1.559 | 0.255 |
| 60-69 | 138 |  |  |  |  |  |
| 70-81 | 125 | 1.659 | 1.235 | - | 2.229 | <0.001 |
| Histologic type |  |  | | | | |
| Differentiated | 136 | 1  1.102 | 0.855 | - | 1.421 | 0.454 |
| Undifferentiated | 237 |  |  |  |  |  |
| Initial Stage |  |  | | | | |
| II | 106 | 1  1.616 | 1.229 | - | 2.126 | <0.001 |
| IIIA | 163 |  |  |  |  |  |
| IIIB | 106 | 1.396 | 1.029 | - | 1.895 | 0.032 |
| Time from surgery to recurrence |  |  | | | | |
| <1 year | 120 | 1  0.644 | 0.498 | - | 0.833 | <0.001 |
| ≥ 1year | 255 |  |  |  |  |  |
| Site of First Relapse |  |  | | | | |
| Local recurrence |  |  |  |  |  |  |
| - | 347 | 1  1.196 | 0.720 | - | 1.984 | 0.489 |
| + | 28 |  |  |  |  |  |
| Lymph node recurrence |  |  |  |  |  |  |
| - | 292 | 1  1.938 | 1.380 | - | 2.723 | <0.001 |
| + | 83 |  |  |  |  |  |
| Peritoneum |  |  |  |  |  |  |
| - | 202 | 1  2.703 | 1.855 | - | 3.938 | <0.001 |
| + | 173 |  |  |  |  |  |
| Hematogenous |  |  |  |  |  |  |
| - | 247 | 1  1.842 | 1.310 | - | 2.590 | <0.001 |
| + | 128 |  |  |  |  |  |

Hazard ratios for covariates of interest and survival after recurrence
